# PTEN deficiency and mutant p53 confer glucose-addiction to thyroid cancer cells: impact of glucose depletion on cell proliferation, cell survival, autophagy and cell migration

**DOI:** 10.18632/genesandcancer.21

**Published:** 2014-07

**Authors:** Federica Morani, Suratchanee Phadngam, Carlo Follo, Rossella Titone, Visa Thongrakard, Alessandra Galetto, Oscar Alabiso, Ciro Isidoro

**Affiliations:** ^1^ Laboratory of Molecular Pathology, Department of Health Sciences, Università del Piemonte Orientale “A. Avogadro”, Novara (Italy); ^2^ Department of Clinical Chemistry, Faculty of Allied Health Sciences, Chulalongkorn University, Bangkok, Thailand; ^3^ Unit of Oncology, Department of Translational Medicine, Università del Piemonte Orientale “A. Avogadro”, Novara (Italy)

**Keywords:** Warburg effect, glucose, autophagy, metabolic stress, PTEN, p53

## Abstract

Proliferating cancer cells oxidize glucose through the glycolytic pathway. Since this metabolism is less profitable in terms of ATP production, cancer cells consume large quantity of glucose, and those that experience insufficient blood supply become glucose-addicted. We have analyzed the response to glucose depletion in WRO and FTC133 follicular thyroid cancer cells, which differ in the expression of two key regulators of the glucose metabolism. WRO cells, which express wild type p53 and PTEN, showed a higher rate of cell proliferation and were much less sensitive to glucose-depletion than FTC133 cells, which are PTEN null and express mutant p53. Glucose depletion slowed-down the autophagy flux in FTC133 cells, not in WRO cells. In a wound-healing assay, WRO cells were shown to migrate faster than FTC133 cells. Glucose depletion slowed down the cell migration rate, and these effects were more evident in FTC133 cells. Genetic silencing of either wild-type PTEN or p53 in WRO cells resulted in increased uptake of glucose, whereas the ectopic expression of PTEN in FTC133 cells resulted in diminished glucose uptake. In conclusion, compared to WRO, FTC133 cells were higher glucose up-taker and consumer. These data do not support the general contention that cancer cells lacking PTEN or expressing the mutant p53R273H are more aggressive and prone to better face glucose depletion. We propose that concurrent PTEN deficiency and mutant p53 leads to a glucose-addiction state that renders the cancer cell more sensitive to glucose restriction. The present observation substantiates the view that glucose-restriction may be an adjuvant strategy to combat these tumours.

## INTRODUCTION

Glucose is an essential precursor for the synthesis of various macromolecules and the main source of the energy needed in the survival and biosynthetic pathways. Normally, in the presence of oxygen, glucose is oxidized through the mitochondrial respiration pathway with the highest rate of production of ATP. However, in highly proliferating cancer cells glucose is preferentially converted into lactate despite the presence of oxygen and functional mitochondria. This aberrant metabolism of glucose, known as the Warburg effect or aerobic glycolysis, is much less convenient in terms of energy gain, and imposes a large consumption of glucose in proliferating cells[1,2]. Vascularization is defective and insufficient in fast growing solid tumours and, consequently, the cells located distant from the blood vessels experience a lack of glucose and oxygen[3]. Under these conditions, cancer cells activate autophagy, a pro-survival lysosomal degradation pathway, and produce large quantity of lactic acid. Autophagy itself promotes the metabolic switch toward glycolysis, thus facilitating cell survival and cell proliferation[4]. In addition, autophagy and glycolysis are also involved in the ‘epithelial-to-mesenchymal’ transition phenomenon that precedes the metastasization[5-7]. Therefore, glucose availability and modulation of autophagy have a great impact on the malignant behaviour of cancer cells.

PTEN and TP53 are the most common deleted or mutated oncosuppressors found in human carcinomas. These two oncosuppressors are key regulators of the glucose metabolism and of autophagy[8-11]. Either PTEN deficiency or p53 mutation has been shown to confer the ability to cancer cells to overcome the metabolic stress caused by hypoxia and nutrients (including glucose) depletion, and to be the driving force for cell proliferation and cell migration[8,9,12,13]. Here, we have tested whether combined PTEN deficiency and p53 mutation indeed confers a metabolic advantage to cancer cells in response to glucose depletion in terms of cell survival, cell proliferation and cell migration, and of autophagy response. As a cell model, we employed the follicular type thyroid cancer cell lines WRO and FTC-133. WRO cells express wild-type PTEN and p53, whereas FTC133 cells are PTEN-deficient and express the anti-apoptotic and anti-autophagic p53R273H mutant[14,15]. We found that FTC133 cells were more active up-taker and consumer of glucose than WRO cells. Consistently, the facilitative glucose transporter GLUT1 was basally expressed at higher level on the plasmamembrane of FTC133 cells than on that of WRO cells. Through genetic manipulations we could demonstrate that both the membrane translocation of GLUT1 and the uptake of glucose are controlled by PTEN and p53. Unexpectedly, FTC133 cells were revealed to be more sensitive to glucose-depletion in terms of cell growth, survival and migration, and of autophagy completion, than WRO cells. The present data indicate that the synergistic effects of combined PTEN deficiency and p53 mutation render the cancer cells glucose-addicted and therefore more sensitive to glucose deprivation.

## RESULTS

### Effect of glucose depletion on PTEN and p53 in WRO and FTC133 cells

The effect of glucose depletion on PTEN and p53 expression was analyzed in WRO and FTC133 thyroid cancer cells. To this end, the cells were cultivated in glucose-containing (2 g/L for RPMI and 4.5 g/L for DMEM, respectively for WRO and FTC133 cells) or in glucose-free standard medium for 24 h and then analyzed by western blotting. Both these cell types present no mutations in the *RAS* and *PI3kCA* genes, while FTC133 cells present the following unique mutations: the R273H P53 mutation and the R130STOP PTEN mutation[14]. FTC133 cells have also been reported to bear a monoallelic deletion of PTEN[15].

**Figure 1:**
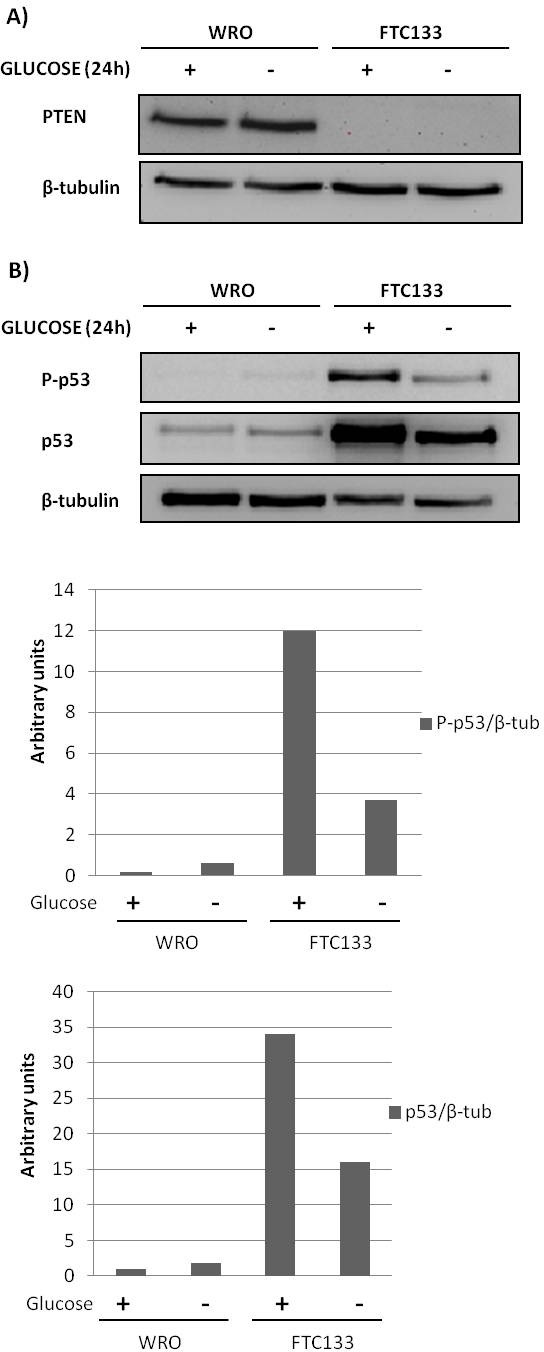
The effect of glucose availability on the expression of PTEN and p53 in WRO and in FTC133 cells WRO and FTC133 cells were plated and let adhere on Petri dishes and then incubated for 24 h in glucose-rich or in glucose-free standard medium. Cell homogenates were analyzed by western blotting for the expression of PTEN, ser15-phosphorylated p53 and total p53 as indicated, respectively in panel A and B. The filters were stripped and re-probed for β-tubulin as a protein loading marker. Densitometry of p53 bands in panel B is included. The blots here shown are representative of n=3 independent experiments. Glucose-dependent difference in the expression of PTEN in WRO cells (panel A) was not statistically significant.

As a consequence of the mutations, PTEN protein was not detectable in FTC133 cells (Figure 1A). In WRO cells, PTEN was expressed at high level and its expression was not subjected to substantial changes in dependence of glucose availability (Figure 1A). The mutant p53 was highly expressed in FTC133 cells, when compared to the expression of the wild-type p53 in WRO cells (Figure 1B). This finding is consistent with literature data on the abnormal hyper-expression of mutant p53 in tumours. Noteworthy, glucose depletion greatly reduced the protein level of the mutant p53 in FTC133 cells, not that of the wild-type p53 in WRO cells (Figure 1B). Phosphorylation of p53 at ser15 stabilizes the protein and is indicative of its activation. In fact, wild-type p53 was phosphorylated and its protein level slightly increased in WRO cells cultivated for 24 h in glucose-free medium. Unexpectedly, a large proportion of the mutant p53 in FTC133 cells was phosphorylated, and about one-third of it was degraded upon 24 h glucose depletion (Figure 1B). These data indicate that WRO and FTC133 cells respond differently to glucose depletion in terms of p53 activation and stability.

### Glucose depletion differentially affects WRO and FTC133 cell proliferation

To determine the effect of glucose depletion on cell proliferation, WRO and FTC133 cells were plated at the same starting density, let adhere for 24 h in glucose-containing complete medium (cell density at this time was considered as t0), then washed and further cultivated in glucose-containing or glucose-free medium for up to 48 h without medium change. Cell density was evaluated at 24 h and 48 h of incubation and the doubling time (Dt) of the cell population was calculated (Table 1). In the presence of glucose, the rate of proliferation (as mirrored by the Dt) in both cell types remained substantially unaltered, indicating that the consumption of nutrients (glucose, aminoacids) in the first 24 h did not affect much the duplication potential in the subsequent 24 h of incubation. Strikingly, the Dt of FTC133 cells was two folds longer than that of WRO cells, and this in spite of the fact that they were cultured in high-glucose medium. When incubated in the absence of glucose, the Dt increased for both cell types, indicating a strict dependence on the availability of glucose for their duplication. However, in WRO the Dt only increased by 1.5-folds (from ~13.5 h to ~22.5 h) while in FTC133 the Dt increased by 3.5-fold (from 27 h to 96 h), i.e. more than two times. The different dependency from glucose for cell duplication became even more evident when evaluated after 48 h of culture in glucose-free medium. Under this condition, the Dt of FTC133 cells was approximately four-times that of WRO cells (230 h *vs* 60 h).

**Table 1:** Doubling time of WRO and FTC133 cells after incubation in glucose-containing complete medium or in glucose-free medium

	24h	48h
WRO +glucose	13.47 ± 1.46	16.33 ± 2.54
WRO -glucose	22.43 ± 3.83	60.09 ± 5.91
FTC133 +glucose	27.08 ± 4.89	26.4 ± 5.05
FTC133 –glucose	95.9 ± 4.47	228.83 ± 0.74

In the presence of glucose the doubling time of FTC133 cells was two-folds longer than that of WRO cells. In glucose depleted culture condition, the doubling time of FTC133 cells increased much more than that of WRO cells, indicating a higher dependence on the availability of glucose for their duplication.

**Figure 2:**
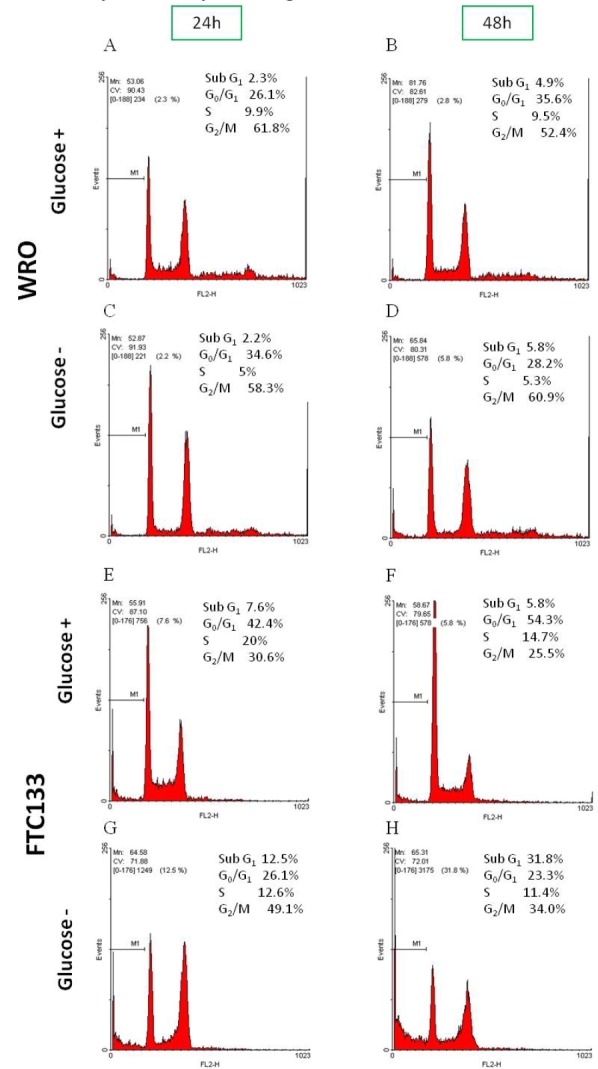
Different growth response to glucose availability between WRO and FTC133 cells WRO and FTC133 cells were let adhere for 24 h in complete medium and then cultivated in glucose-containing or in glucose-free standard medium for up to 48 h without medium change. At the end, adherent and suspended cells were recovered, fixed in ethanol and labelled with propidium iodide (PI). Finally, the cells were analyzed by cytofluorometry to determine the phases of cell cycle. In panels A-H are shown the percentages of the four cell cycle phases (Sub G1, G0/G1, S, G2/M) obtained using the software Win MDI 2.9. During glucose depletion WRO cells accumulated in the G0/G1 retarding the entry in the S phase (panels C-D *vs* A-B) and only a small fraction of the cells underwent apoptosis (panel D); FTC133 cells in the first 24 h accumulated in G2/M and underwent apoptosis (indicated by subG1 peak, panels G), while in the following 24 h decreased the fraction of cells in G2/M and increased the fraction of subG1 population (panel H *vs* G). Data shown in this Figure have been reproduced independently four times.

The difference in the response to glucose availability between the two cell lines was further substantiated by the cell cycle analysis (Figure 2). Between 24 h and 48 h, WRO cells cultivated in the presence of glucose slightly increased the fraction in G0/G1, while not changing the amount in the S phase (panel B *vs* A). These data are in agreement with the substantial similar Dt calculated at 24 and 48 h (see Table 1). When cultivated in the absence of glucose, WRO reduced the fraction in the S phase and tended to accumulate in the G0/G1, indicating that the lack of glucose retarded the entry in the S phase (panel C *vs* A). In the following 24 h of culture without glucose (panel D *vs* C) no changes were observed, besides the fact that a small fraction (4%) of the WRO cells resting in G0/G1 underwent apoptosis (as indicated by the apparent increase of the subG1 peak).

Between 24 h and 48 h in glucose-containing medium, FTC133 cells reduced their fraction in S and G2/M phases to accumulate in the G0/G1 phase, likely as a consequence of the consumption of nutrients in the first 24 h, but no increase of the SubG1 fraction was recorded (panel F *vs* E). When cultivated for 24 h in the absence of glucose, FTC133 cells reduced their fraction in the G0/G1 and S phases, and tended to accumulate in the G2/M phase and also to undergo apoptosis (as indicated by the increase in the subG1 peak), suggesting that the lack of glucose could have impaired the completion of the mitotic process (panel G *vs* E). In the following 24 h of culture without glucose the above effect was even more evident, as the fraction of FTC133 cells in G2/M further decreased and correspondingly increased the fraction in the subG1 population (panel H *vs* G).

Overall, these data demonstrate that FTC133 are much more sensitive than WRO cells to nutrient shortage, especially to glucose starvation.

### Glucose depletion differentially affects WRO and FTC133 cell survival

**Figure 3:**
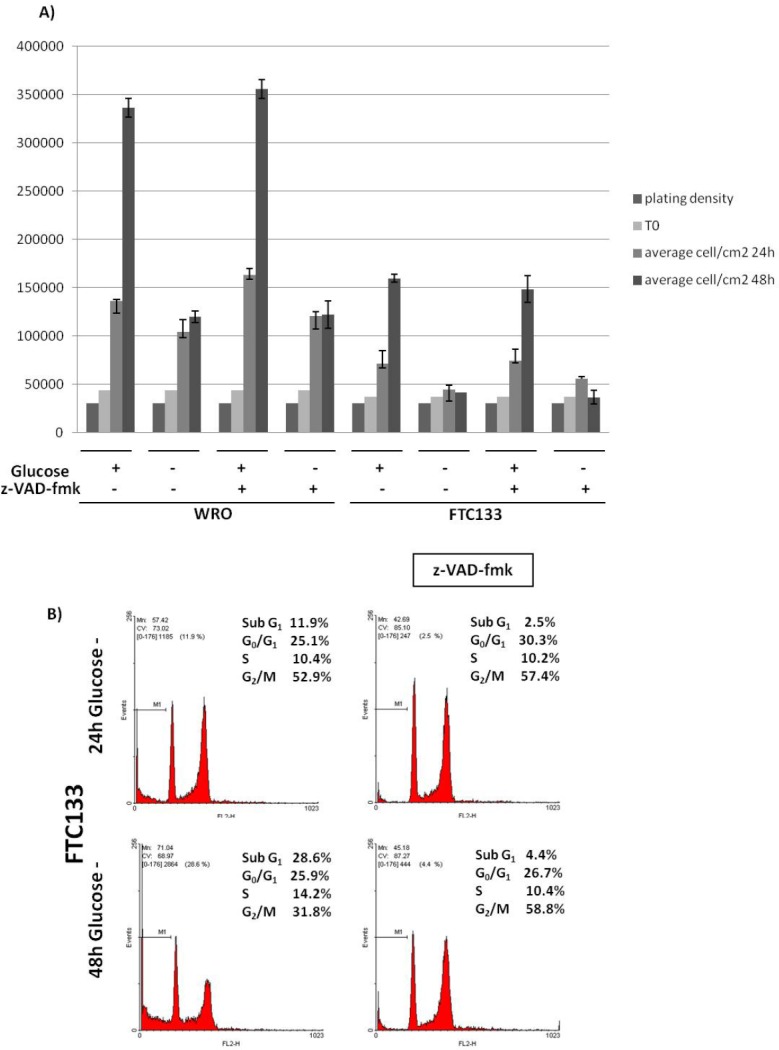
Glucose depletion induces apoptotic cell death in FTC133 cells A) WRO and FTC133 cells were cultivated in glucose-containing or in glucose-free standard medium for up to 48 h without medium change in the absence or presence of the pan-caspase inhibitor z-VAD-fmk, and then counted. In glucose depletion condition, no apoptosis occurred in WRO cells, while in FTC133 cells apoptotic cell death occurred at 24 h (partially rescued by z-VAD-fmk). B) Cytofluorometry analysis of the cell cycle in FTC133 cells cultivated in glucose-free condition for up to 48 h in the absence or presence of the pan-caspase inhibitor z-VAD-fmk confirmed the induction of apoptotic cell death at 24 h. Z-VAD-fmk prevented apoptosis in the first 24 h and arrested the cell cycle in the G2/M phase, avoiding necrosis, in the following 24 h (48 h). Data shown in this Figure have been reproduced independently four times.

To assess whether glucose depletion caused apoptosis to FTC133 cells, we repeated the experiment in the presence of the pan-caspases inhibitor z-VAD-fmk. No apoptosis occurred in WRO cells cultivated under any conditions, while in the case of FTC133 cells cultivated in the absence of glucose a portion of cell loss could be rescued by z-VAD-fmk at 24 h, but not at 48 h (Figure 3A). The latter finding was in apparent contrast with the data shown in Figure 2H. However, a cytofluorometric analysis of the cell cycle confirmed that in fact z-VAD-fmk effectively reduced the subG1 peak (indicative of apoptosis) while increasing the fractions in the G2/M phase (Figure 3B). Consistently, in the presence of z-VAD-fmk the percentage of FTC133 cells positively stained with trypan blue, a dye that monitors necrosis, decreased from approximately 18% to 7% in the 48 h culture in glucose-free medium. Thus, in FTC133 cells cultivated for 48 h in the absence of glucose, z-VAD-fmk prevented apoptosis in the first 24 h, while in the subsequent 24 h it caused a general block of the cell cycle that prevented the onset of necrosis.

### Glucose availability differentially impact autophagy in WRO and FTC133 cells

The autophagy-lysosomal degradation pathway is up-regulated under stressful metabolic conditions to provide the cells with anabolic substrates necessary for survival[16]. Nutrients (essentially aminoacids and glucose) shortage, as well as the lack of growth factors, are strong stimuli for rising up basal autophagy to the level needed to overcome the metabolic stress[16]. The altered expression of PTEN and p53 is likely to affect the regulation of autophagy in thyroid cancers[17].

**Figure 4:**
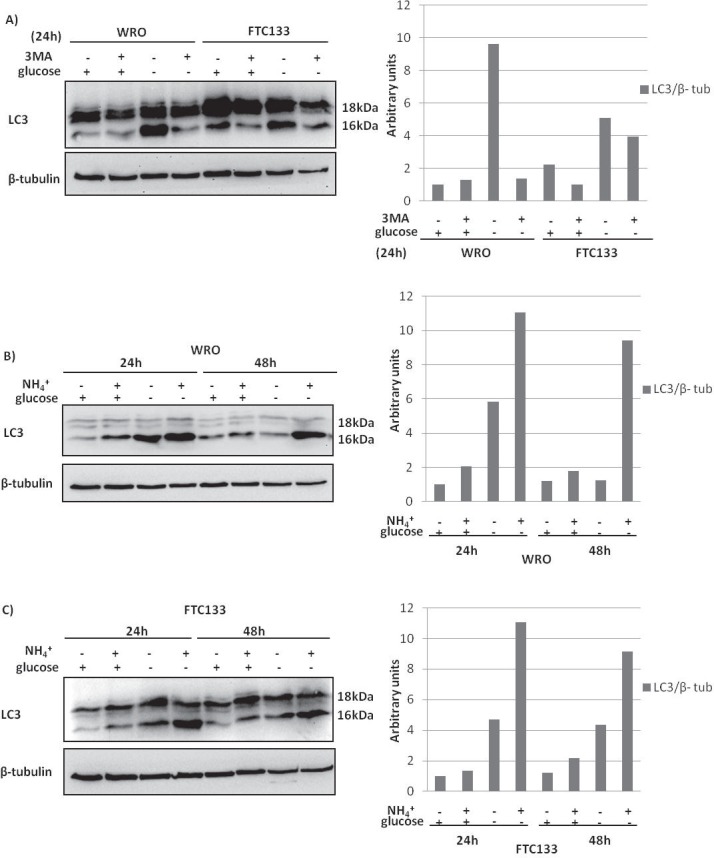
Induction of autophagy in WRO and FTC133 cells by glucose depletion A) Western blotting analysis of the autophagosome marker LC3 II in WRO and FTC133 cells cultivated for 24 h in glucose-containing or in glucose-free standard medium in the absence or the presence of the PI3k inhibitor 3MA (10mM). The densitometry ratio of LC3 II normalized *versus* β-tubulin is reported. 3MA inhibited glucose-depletion induced autophagy more in WRO cells than in FTC133 cells. B-C) Western blotting analysis showing LC3 II levels in WRO cells (B) and in FTC133 cells (C) cultivated for 24 h and 48 h in glucose-containing or in glucose-free standard medium in the absence or the presence of the weak base ammonium chloride (NH_4_Cl, 10mM). The densitometry ratio of LC3 II normalized *vs* β-tubulin is reported. Under glucose depletion autophagy induction appeared similar in the first 24 h for both the cell lines, instead at 48 h the autophagy flux was impaired in FTC133 cells. Data shown in this Figure have been reproduced independently three times.

It seemed therefore important to check whether the different susceptibility to glucose deprivation manifested by WRO and FTC133 cells could be associated with an altered activation of the autophagy pawthay. During autophagosome formation, LC3 must be post-translationally conjugated to phosphatydil-ethanolamine in order to be inserted onto the autophagosomal membranes. This processing increases the apparent electrophoretic mobility of LC3, thus making easy in western blotting to distinguish the precursor LC3-I (apparent m.w. of 18 kDa) and the mature isoform LC3-II (apparent m.w. of 16 kDa). In the canonical regulatory pathway of autophagy, PI3k class III (also known as Vps34) provides the essential starting signal for the autophagosome formation. We included in our incubation conditions 3-methyl adenine (3MA), an inhibitor of PI3k. This drug is widely employed as a pharmacological inhibitor of autophagy[18], though it has been reported that depending on the cell line and the dose and time of incubation it might elicit a paradoxic induction of autophagy[19]. Based on the assumption that the cellular level of LC3-II reflects the autophagosomes present in the cells, it appears that in standard culture condition autophagy is basally (2.5-folds) higher in FTC133 than in WRO cells (Figure 4A). 3MA inhibited only partially basal autophagy in FTC133 cells, not in WRO cells. Glucose depletion induced autophagy in both cell lines, yet the response was higher in WRO than in FTC133 cells (the increases of LC3-II in glucose-deprived samples were of 10-folds and of 2.2-folds above the controls, respectively). Of note, 3MA effectively inhibited glucose-deprivation induced autophagy in WRO cells, and only slightly in FTC133 cells. The increase of LC3-II *per se* not necessarily proves the induction of autophagy, since it might also simply represent the accumulation of autophagosomes resulting from the block of their consumption within the lysosomes[18]. To discriminate true autophagosome production from autophagosome accumulation, it is useful to artificially impair the fusion and degradation steps by using a lysosomal pH disruptor such as ammonium chloride[18]. In the following experiments, we assessed the amount of LC3-II in the cells incubated in the presence of ammonium chloride. Under standard (glucose-containing) culture condition, basal autophagy runs at higher level in WRO than in FTC133 cells, as indicated by the higher accumulation of LC3 II in the presence of ammonium chloride (Figure 4B). At 48 h (with no change of medium), only FTC133 cells have increased the production of autophagosomes. In the absence of glucose, autophagy is induced both in WRO and FTC133 cells and the production/consumption rates of autophagosomes (based on LC3 II accumulation in the absence/presence of ammonium chloride) appear similar in the first 24 h. However, at 48 h WRO cells do not produce new autophagosomes, and rather consume all the pre-existing ones, whereas FTC133 cells still accumulate a large part of the pre-existing autophagosomes indicating that the last step of the autophagy flux is impaired. The above data were further corroborated by immunofluorescence staining of LC3-positive vacuoles (not shown).

### Glucose depletion differentially affect WRO and FTC133 cell migration

**Figure 5:**
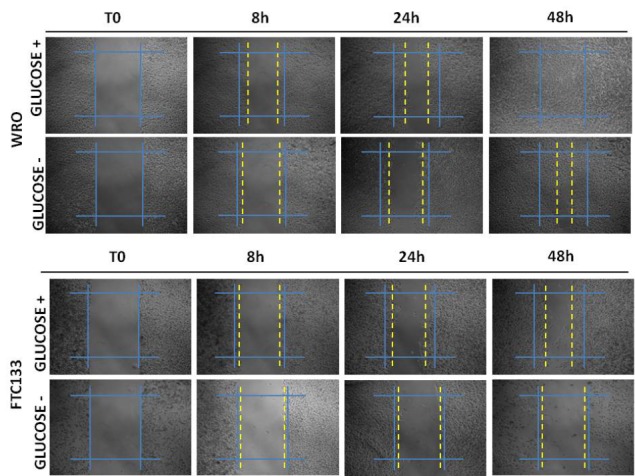
Glucose depletion differentially affect cell migration of WRO and FTC133 cells WRO and FTC133 cells were plated and let grow to confluence in Petri dishes in glucose-containing standard medium, then a scratch-wound was made using a tip and the cell cultures were switched into glucose-containing or glucose-free conditions and cultured for up to 48 h. Photographs of the wound were taken at time 0 and at 8, 24 and 48 h. The wound area and the percentage of healing were calculated for each time point. WRO cells showed a higher migration rate than FTC133 cells. Data shown in this Figure have been reproduced independently three times.

**Figure 6:**
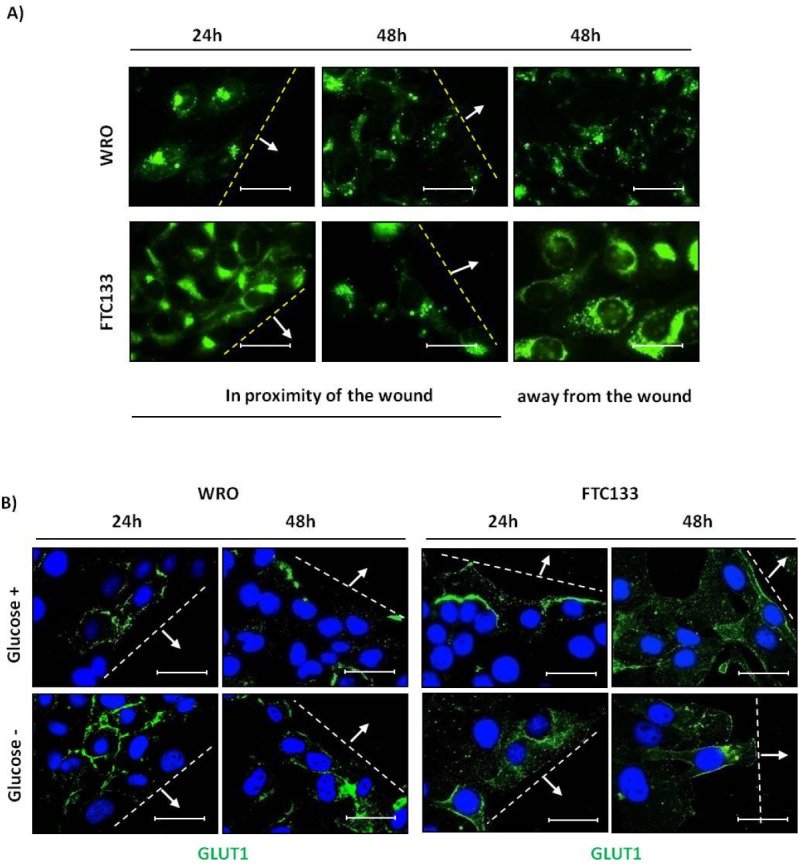
Differential uptake of glucose and plasmamembrane expression of GLUT1 in WRO and FTC133 cells A) Glucose uptake assay in WRO and FTC133 cell lines. The uptake of the glucose fluorescent analogue 2-NBDG (in green, 50 μM) was monitored during glucose deprivation in proximity of the wound or far from the wound at 24 h and 48 h. Level of 2-NBDG uptake was higher in FTC133 cells than in WRO cells. Scale bar = 20μm. Magnification= 63X. B) Plasmamembrane expression of GLUT1 in proximity of the wound in WRO and FTC133 cell lines. Cells grown on coverslips were cultivated in glucose-containing or glucose-free culture medium for 48 h as per the wound healing assay and immunostained for GLUT1. The expression of GLUT1 in plasmamembrane was higher in FTC133 cells than in WRO cells. In WRO cells the membrane expression of GLUT1 increased under glucose deprivation. Nuclei are stained with DAPI. Scale bar = 20μm. Magnification= 63X. Data shown in this Figure have been reproduced independently three times.

To assay the influence of glucose availability on the migratory potential of thyroid cancer cells, we tested the effect of glucose deprivation in a classical scratch-wound healing assay[20]. WRO cells showed a higher migration rate than FTC133 cells (Figure 5). In glucose-containing medium condition, WRO cells completely healed the wound by 48 h, whereas at this time FTC133 cells only covered a 50% of the wound area. Under glucose-deprivation condition, cell migration was much slower, yet this effect was particularly evident in FTC133 cells which essentially stopped their migration from 24 h on (Figure 5, Table 2). To get an insight on the functional link between glucose availability and cell migration, we monitored the uptake of glucose in migrating WRO and FTC133 cells in the proximity of the wound. For this purpose, we used 2-(N-(7-Nitrobenz-2-oxa-1,3-diazol-4-yl)Amino)-2-Deoxyglucose (2-NBDG), a fluorescent analogue of glucose widely used for optical measurements of glucose uptake[21]. The uptake of the fluorescent probe was better appreciated in the cells cultured in glucose-free medium, given that 2-NBDG competes with glucose for the same membrane transporter of the GLUT family. In this condition, the 2-NBDG fluorescent signal was much more intense in FTC133 cells than in WRO cells at any time-point, either in the vicinity of the wound and in the rear (Figure 6A). Thus, despite of the fact that cell proliferation and cell migration were much slower, FTC133 cells appeared more avid than WRO cells in the uptake of glucose. Glucose is internalized in the cells by glucose transporters belonging to the GLUTs family[22]. GLUT1 is the most prevalent isoform in highly aggressive and less-differentiated thyroid cancer histotypes[23-25]. We checked the plasmamembrane expression of GLUT1 in migrating WRO and FTC133 cells cultivated in glucose-containing or in glucose-free culture medium. Based on immunofluorescence staining, it is apparent that FTC133 cells express GLUT1 on the plasmamembrane at level higher than in WRO cells (Figure 5B). It is to note that in WRO cells the membrane expression of GLUT1 increases upon incubation in glucose-free medium (Figure 6B).

Table 2Percentage of healing in WRO cells (A) and in FTC133 cells (B) in glucose-containing or glucose-free condition at 8 h, 24 h, and 48 h

**A):** 

	WRO	
	+ Glucose	− Glucose
8h	33.30%	16.60%
24h	50%	27.70%
48h	100%	66.60%

**B):** 

	FTC133	
	+ Glucose	− Glucose
8h	20.00%	10.00%
24h	35%	15.00%
48h	50%	15.00%

Migration was quantified by calculating the area of the wound at the different time points. Cell migration was slower in FTC133 cells than in WRO cells, and in the absence of glucose migration was further reduced.

### PTEN and p53 control the membrane expression of GLUT1 and the uptake of glucose in thyroid cancer cells

**Figure 7:**
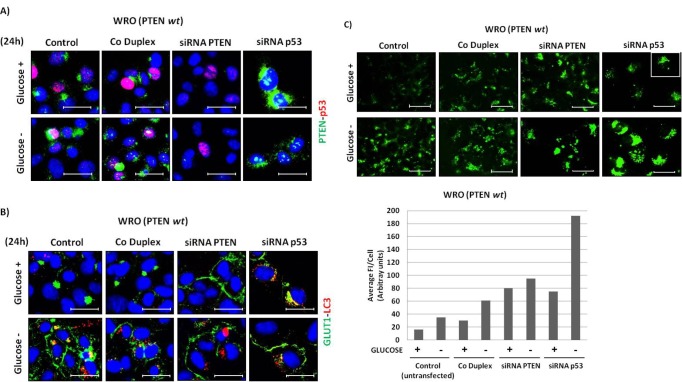
Effect of PTEN and p53 gene knock-down on the cellular expression of GLUT1 and LC3 and on glucose uptake in WRO cells WRO cells plated on coverslips were transfected with control duplex (sham) or with siRNA specifically targeting PTEN or p53. Control un-transfected cells were also included. The cells were then incubated for 24 h in glucose-containing or glucose-free medium. At the end, the coverslips were processed for immunofluorescence staining. A) co-immunostaining of PTEN and p53; B) co-immunostaining of GLUT1 and LC3. C) A parallel set of cultures was used to assay the uptake of 2-NBDG. ImageJ quantification of cell associated 2-NBDG is included. Data are given as average of Fluorescence Intensity (FI) per cell in the selected fields. These data were reproduced independently two times in double. Scale bar = 20μm. Magnification= 63X.

To definitively link PTEN and p53 to the capability of the cancer cells to uptake glucose, we sought to manipulate genetically the expression of these proteins. First, we employed the small interference technology to silence the expression of wild-type PTEN and of wild-type p53 in the WRO cells. After trasnfection, the cells were cultivated for 24 h in the presence or absence of glucose. As shown by immunofluorescence staining in Figure 7A, the transfection with the specific siRNA achieved the effective down-regulation of either PTEN or p53 in the large majority of the cells (it was estimated that >80% of the cells in the monolayer were negative for the protein considered). It is to be noted, however, that the silencing of p53 was of detriment for cell survival (it was estimated that about 40% of the transfected WRO cells were detached at the end of the incubation period). This toxic effect was exacerbated in the glucose-deprived culture. Next, we performed in a parallel set of cultures the immunostaining for GLUT1 and for LC3. In control un-transfected and in sham-transfected cells, GLUT1 clustered in a para-golgian area or was relocated at the plasmamembrane depending on whether the cells were cultivated in the presence or the absence of glucose (Figure 7B). To be noted, the cells transfected with either the siRNA specific for PTEN or for p53 basally showed GLUT1 on the plasmamembrane, regardless of the presence or absence of glucose in the culture medium (Figure 7B). LC3-positive autophagic vacuoles were clearly detected in a perinuclear region of the cells cultivated in the absence of glucose, consistent with induction of autophagy by glucose deprivation (see Figure 5B). Of note, LC3-positive vacuoles were also detected in the cells transfected with siRNA p53 cultivated in the presence of glucose.

We then investigated on the functional consequences of the PTEN or p53 knock-down in terms of glucose uptake. The images shown in Figure 7C clearly demonstrate that either the silencing of PTEN or of p53 greatly favoured the uptake of glucose. By ImageJ quantification it was estimated that the uptake of 2-NBDG in siRNA-transfected cells was on average three folds that in the sham-transfected counterpart and five to six folds that in the un-transfected control cells (Figure 7C, lower panel). In siRNA p53-transfected cells cultivated in the absence of glucose the uptake of 2-NBDG was even higher. Note that the stress associated to the transfection *per se* stimulated the uptake of 2-NBDG (compare sham-transfected *vs* un-transfected).

**Figure 8:**
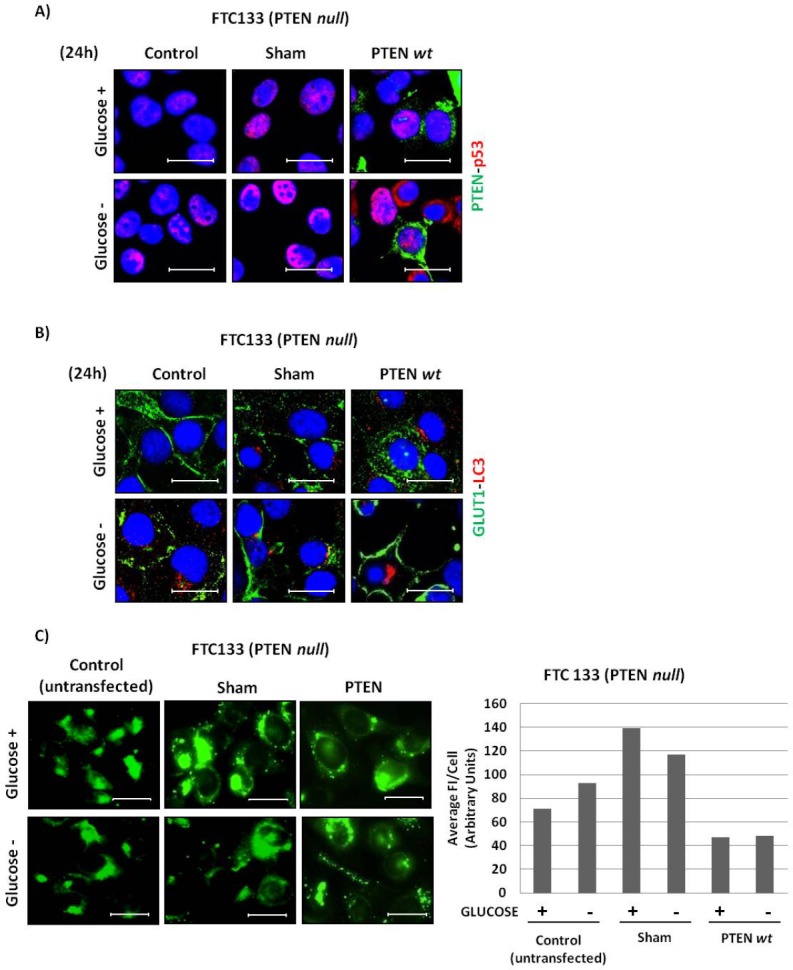
Effect of ectopic expression of PTEN in FTC133 cells on the cellular expression of GLUT1 and LC3 and on glucose uptake FTC133 cells plated on coverslips were transfected with an empty pcDNA vector (sham) or with the plasmid harbouring the wild-type PTEN cDNA. Control un-transfected cells were also included. The cells were then incubated for 24 h in glucose-containing or glucose-free medium. At the end, the coverslips were processed for immunofluorescence staining. A) co-immunostaining of PTEN and p53; B) co-immunostaining of GLUT1 and LC3. C) A parallel set of cultures was used to assay the uptake of 2-NBDG. ImageJ quantification of cell associated 2-NBDG is included. Data are given as average of Fluorescence Intensity (FI) per cell in the selected fields. These data were reproduced independently three times. Scale bar = 20μm. Magnification= 63X.

Finally, as a complementary experiment to prove the role of PTEN in glucose uptake, we transgenically expressed wild-type PTEN in the PTEN-null FTC133 cells. After transfection with a plasmid harbouring the PTEN cDNA, the cells were further incubated 24 h in glucose-containing or glucose-free medium. A first set of coverslip was immunostained for PTEN and p53 (Figure 8A). Based on immunofluorescence positivity it was estimated that >60% of the transfected cells were efficiently expressing PTEN. Of note, the mutant p53 protein localized to the nucleus, especially in the cells cultivated in glucose-free medium (Figure 8A). In control un-transfected and in sham-transfected FTC133 cells GLUT1 was permanently localized on the plasmamembrane, regardless of the availability of glucose (Figure 8B). The ectopic expression of PTEN greatly compromised the membrane localization of GLUT1 (Figure 8B). Yet, when cultivated in the absence of glucose GLUT1 was relocated again on the plasmamembrane (Figure 8A). We then looked at the consequence of PTEN expression on the uptake of glucose in transfected FTC133 cells. Strikingly, the fluorescence associated with the internalization of 2-NBDG was drastically reduced in the PTEN-transfected cultures (Figure 8C). By ImageJ quantification, the uptake of 2-NBDG was halved compared to that of control un-transfected cells, regardless of whether the cells were cultivated in the presence or the absence of glucose (Figure 8C, right panel). This effect is likely underestimated if one considers that the transfection *per se* stimulated the uptake of the fluorescent probe (compare the sham-transfected with the un-transfected cells) and that not all the transfected cells expressed PTEN at high level.

From these data we can conclude that PTEN and p53 control the membrane expression of GLUT1 and the uptake of glucose in cancer cells.

## DISCUSSION

In solid tumours, glucose and oxygen availability decrease in the most inner portion as increases the distance of cancer cells from the peripheral vasculature[3]. Hypoxic tumour cells are more aggressive and metastatic-prone, as they reprogram their metabolism toward aerobic glycolysis through HIF-1α-mediated expression of GLUT proteins and glycolytic enzymes[22,26]. Hypoxia has been shown to induce a metabolic response, mediated by the Hypoxia Inducible Factors (HIF) 1 and 2 alpha, that promotes the migration and spreading of thyroid cancer cells[27,28]. Interestingly, this behaviour was reinforced by the abnormal activation of the PI3k pathway in cells lacking the expression of PTEN[28]. It has also been reported that glycolysis is a main source of energy for malignant thyroid cells[29]. However, no studies have so far analyzed the effect of glucose depletion on the phenotypic behaviour of thyroid cancer cells. Here we have addressed this issue in the thyroid cancer cell lines WRO and FTC133, which differ in the expression of PTEN and p53, two oncosuppressor proteins that are known to play important roles in glucose metabolism. PTEN hyper-expression reduces glucose uptake and favours its mitochondrial oxidation, thus opposing the Warburg effect[8]. Further, PTEN down-regulates the PI3k-AKT-mTOR pathway, that transduces cell proliferation signals[30]. TP53 also reduces the glucose uptake and antagonizes the Warburg effect by controlling the expression of membrane glucose transporters[31] and of glycolytic enzymes[32]. Activation of p53 also leads to cell cycle arrest[33] and apoptosis[34]. Therefore, either loss of PTEN or of p53 is expected to increase glucose uptake and glycolysis, to promote cell proliferation and to impair apoptosis. Indeed, we found that FTC133 cells, which are PTEN deficient and express a mutant p53, are higher up-taker and consumer of glucose respect to WRO cells, which express wild-type PTEN and p53. Experiments of (PTEN and p53) gene silencing and of (PTEN) transgenic expression clearly demonstrated the dominant role of PTEN and p53 in driving the plasmamembrane localization of GLUT1 and the glucose uptake. Worth to note, while the wild-type p53 was stabilized in glucose-depleted WRO cells, the mutant p53 was instead degraded in glucose-depleted FTC133 cells despite it was phosphorylated. Wild-type p53 is mainly degraded through the MDM2-ubiquitin-proteasome system[35], while the mutant p53 is degraded by either the chaperon-mediated autophagy[36] or the macro-autophagy pathway[37]. Also to be noted is that knock-down of wild-type p53 in WRO cells was deleterious for cell survival, while increasing basal autophagy and glucose uptake in the cells. Knock-down of PTEN increased the rate of glucose uptake in WRO cells, and it had no side effects on cell viability and on the accumulation of LC3-vacuoles.

Strikingly, the Dt of FTC133 cells was two-folds that of WRO cells when cultured in glucose-containing medium, and it further increased to four-folds when cultured for up to 48 h in glucose-free medium. Cell cycle analysis data indicated that glucose deprivation mainly affected the Go/G1 to S phase transition in WRO cells, though not toxic, while it dramatically affected the completion of the G2/M phase and eventually provoked apoptotic cell death in FTC133 cells. FTC133 expressed high level of plasmamembrane GLUT1, compared to WRO cells, which is consistent with the loss of function of PTEN and p53. FTC133 cells, besides being PTEN-deficient, express the p53R273H mutant that has been shown to inhibit caspase-dependent apoptosis[38] and autophagy[39] and to promote cell migration and invasion[40]. Therefore, it appears counterintuitive that prolonged glucose depletion could lead FTC133 to caspase-dependent cell death. Not only, FTC133 cells also showed a reduced migration rate compared to WRO cells, and this difference was more evident in glucose-depleted conditions. Autophagy was not inhibited in FTC133, yet chronic glucose-depletion led to impaired consumption of the autophagosomes, likely as a consequence of insufficient energy. Taken together, these facts indicate that FTC133 cells rapidly consume glucose, thus soon becoming glucose-addicted. In this respect, it is to be stressed that FTC133 cells were cultivated in a medium containing much more glucose than that used for culturing WRO cells (4.5 g/L *vs* 2 g/L).

We hypothesize that the synergistic effects of combined PTEN loss-of-function and mutant p53 gain-of-function lead to a metabolic dependence on glucose with unpredicted impact on cell behaviour, that differs from that described for individual oncosuppressor gene alteration. Of particular relevance is the impairment of the autophagy flux in glucose-depleted cells, which may account for loss of protection against the metabolic stress.

It is now clear that the malignant behaviour of cancer cells results from the combination of mutations with gain-of-function or loss-of-function that involve at least five to seven oncogenes and oncosuppressor genes. In addition, epigenetic events contribute to the altered expression of key regulatory proteins. The presence of pro-oxidant molecules and of inflammatory cytokines in the tumour micro-environment further contributes to modify the gene expression in cancer cells. Thus, cancers that develop spontaneously soon become a very heterogeneous population of subclones each with its unique set of alterations in the expression of oncogenes and oncosuppressors, as well as of key metabolic regulators. This explains why a drug targeting one single pathway, while effective in experimental models (in which only one gene is the dominant driver), often fails when translated into the clinic for the therapy of spontaneous tumours.

Whatever the set of genes altered, for their proliferation and migration the cancer cells need energy and this depends on the availability of oxygen and nutrients such as glucose and aminoacids (among which glutamine is the most important). Thus, targeting the energetic metabolism of cancer cells may be the clue to revolutionize cancer treatment[41-43]. In particular, glucose availability and glycolysis have been shown causally linked to chemoresistance, proliferation and metastasization of tumours[44-46]. Therefore, glucose-restriction is expected to elicit beneficial effect against glucose-addicted cancer cells. Indeed, there are indications in this sense. Reducing the uptake of glucose with the GLUT1 inhibitor Phloretin favoured doxorubicin toxicity in P-glycoprotein expressing chemoresistant cancer cells[47]. In addition, the administration of 2-deoxy-D-glucose, a glucose analogue that impairs glycolysis, enhanced the clinical efficacy of radio- and chemotherapy in glioblastomas[48] and in anaplastic thyroid cancer[49]. Consistent with the role of glucose availability in cancer progression, it was found that a higher dietary glucose intake significantly associated with an increased risk of recurrence and mortality in stage III colon cancer patients[50].

Here we have shown that the concurrent deletion of PTEN and mutation of p53 exacerbates glucose uptake and consumption in FTC133 cancer cells, which undergo cell cycle arrest and apoptosis, cell migration arrest and manifest defective autophagy when subjected to glucose-restriction. Therefore, targeting together the PI3k-(PTEN)-AKT-mTOR and the p53 pathways, that control both glucose uptake and autophagy, could be an efficacious strategy to cure aggressive cancers.

## MATERIALS AND METHODS

### Cells and treatments

The WRO and FTC133 follicular thyroid cancer cell lines were kindly provided by Dr. Francesco Frasca, University of Catania, Italy. WRO cells were cultured in RPMI 1640 (with L-glutamine) completed by foetal bovine serum (FBS, 10%) and penicillin/streptomycin (1%); the FTC133 cells were cultured in Dulbecco's Modified Eagle Medium, Nutrient mixture F-12 (1:1, by volume) completed by FBS (10%), penicillin/streptomycin (1%) and L-glutamine (1%). All culture reagents were purchased from Sigma-Aldrich (Germany). For studies on glucose deprivation, the cells were incubated in complete culture medium or medium without glucose (R1383 and D5030, Sigma-Aldrich) for up to 48h. For the experiments, growing cells were plated on sterile plastic dishes or on sterile glass coverslips and allowed to adhere for at least 24 h before the use. The PI3K inhibitor 3-methyladenine (3MA; Sigma-Aldrich) was used at 10 mM. Ammonium Chloride (NH_4_Cl) was used at 10 mM. At the end of the incubations, the cells or coverslips were collected and processed as detailed below.

### Cell proliferation, cell cycle and cell death assay

Cell growth was assessed by manual and hemocytometer cell counting of adherent viable (trypan blue-excluding) cells. Doubling Time (Dt) was calculated using the free software Doubling Time Online Calculator (http://www.doubling-time.com/compute.php). Cell death was assessed by counting the trypan blue-stained cells (necrotic cells) and by cytofluorometric analysis of Annexin V-Propidium Iodide double stained cells, as previously reported[51,52]. For this purpose, floating (dead) cells and attached cells were combined. Cell cycle analysis was performed by cytofluorometry of Propidium Iodide labelled cells using the software WinMDI 2.9.

### Wound-healing migration assay

The cells were plated on Petri dishes and cultured for at least 48 h till confluence. A wound was made by dragging a sterile blue pipette tip along the centre of the plate[53]. Detached cells were washed out twice, and cultures were then incubated for up to 48 h with no changes of medium in Glucose-containing or Glucose-free medium. Images of cell monolayers were taken at the time indicated under the phase-contrast microscope with a digital camera. The wound wideness was calculated by measuring the mean distance between the margins of the wound in randomly selected fields, directly on photographs. Migration was quantified by calculating the area of wound at time points *t*_0_(time of wound), *t*_24_ (24 h after wound) and *t*_48_ (48 h after wound). Normalization was obtained by the formula [area(*t*_0_) − area(*t*_24 or 48_)]/area(*t*_0_). Overall, four independent experiments were performed.

In other experiments, the cell were cultured up to confluence on sterile glass coverslips and wounded as described above, except that a yellow pipette tip was used. At the end of the incubations, the cells on coverslips were fixed, permeabilized and processed for immunofluorescence staining. Alternatively, the living cells were assayed for glucose uptake.

### Glucose uptake assay and measurement

The fluorescent glucose analogue 2-[N-(7-nitrobenz-2-oxa-1,3-diazol-4-yl)amino]-2-deoxy-d-glucose (2-NBDG; Life Technologies Co, Carlsbad, CA, USA) was used to detect glucose uptake in living cells[20]. Cells grown on coverslips were incubated with 50 μM of 2-NBDG for 1 h before the end of the treatments, washed twice with PBS 1x and rapidly imaged under the fluorescence microscope. Image processing and data quantification of the area and of the intensity of fluorescence images were performed with the software ImageJ 1.48v (http://imagej.nih.gov/ij/). At least five randomly chosen fields for a total of minimum 50 cells were analysed. Fluorescence Intensity (FI) is given in arbitrary units as an average value per cell in the selected representative fields.

### Immunofluorescence staining

The cells adherent on sterile glass coverslips were fixed in cold methanol for 20 min and permeabilized with 0.2% Triton X-100 in PBS1X for 10 min. Overnight incubation in cold room was performed with the following primary antibodies: rabbit polyclonal anti-GLUT1 (Millipore, Darmstadt, Germany; dilution 1:50); rabbit polyclonal anti-PTEN (Millipore; dilution 1: 200); mouse monoclonal anti-p53 (Santa Cruz Biotechnology, Santa Cruz, CA; dilution 1:100) and mouse monoclonal anti-LC3 (nanoTools, Teningen, Germany; dilution 1:100). As secondary antibody (dilution 1:600) the IRIS-2 (green fluorescence)-conjugated goat-anti-rabbit IgG secondary or IRIS-3 (red fluorescence)-conjugated goat-anti-mouse IgG (Cyanine Technologies SpA, Turin, Italy) was used as appropriate for 1 h at room temperature in a humid chamber. Nuclear chromatin was stained with the fluorescent dye 4,6-diamidino- 2-phenylindole-dihydrochloride (DAPI). As negative control, the primary antibody was omitted or substituted with pre-immune antiserum. Antibodies were diluted in PBS containing 0.1% Triton X-100 and 10% FBS. Stained cells were mounted with Slow-FADE (Light AntiFADE Kit, Molecular Probes Invitrogen, Carlsbad, CA, USA) and observed under a fluorescence microscope (Leica DMI6000B, Leica Microsystem AG, Wetzlad, Germany).

### Western blotting assay

Immunoblotting was performed following standard procedures as previously reported.^54^ Cell homogenates were prepared by freeze–thawing and ultrasonication in a buffer containing detergents and protease inhibitors. 30 μg of cell proteins were denatured with Laemmli sample buffer, separated by electrophoresis on a 12.5% SDS-containing polyacrylamide gel and then electroblotted onto PVDF membrane (Carlo Erba reagents, Milan, Italy). The filter was first probed with the antibody specific for the protein of interest. The following primary antibodies were used: Rabbit polyclonal anti-PTEN (EX-BIO, Vestec, Czech Republic); rabbit polyclonal anti-LC3 (Sigma-Aldrich); rabbit polyclonal anti-phospho p53 (Ser15) (Cell Signaling technology, Danvers, Massachusetts, USA) and mouse monoclonal anti-p53 (Santa Cruz Biotechnology). The filter was subsequently stripped and re-probed with an antibody specific for β-tubulin (Sigma-Aldrich), as an index of homogenate protein loading in the lanes. Immunocomplexes were revealed by using a peroxidase-conjugated secondary antibody (Bio-Rad, California, USA), as appropriate, and subsequent peroxidase-induced chemiluminescence reaction (PerkinElmer, Massachusetts, USA). Densitometry of Western Blot bands was performed with the Quantity One-4.5.0 software (Bio-Rad) and with the free software Image J (1.48v; http://imagej.nih.gov/ij/).

### Small interference RNA and plasmid transfections

The reagents and the methods have been previously described[55]. Post-transcriptional gene silencing was obtained using a specific small interference RNA (siRNA) directed against the mRNA of PTEN (the sense strand was 5′–AGACUUGAAGGCGUAUACA-3′) or of p53 (the sense strand was 5'-AAGAAACCACUGGAUGGAGAAUAUUUC-3'). A control duplex was used for sham-transfection. Duplexes of nucleotide siRNA were purchase from MWG Biotech AG (Ebersberg, Germany). In brief, the cells were plated and let adhere on coverslips for at least 24 h, then incubated for 6 h with 30 pmol RNA-duplexes in the presence of 7.5 μl Lipofectamine 3000 (Invitrogen Co) diluited in 250 μl of Optimem (Life Technologies Co). The cells were then washed and further incubated 36 h post-transfection to allow maximal effect on protein down-regulation before use.

Transgenic expression of *wild type* PTEN was obtained by Lipofectamine 3000 (Invitrogen Co) transfection with a pcDNA3.1Zeo+ plasmid containing the specific full-length cDNA. An empty pcDNA3.1Zeo+ plasmid was used for sham transfection. In brief, the cells adherent on coverslips were incubated for 6 h with 5 μg/μl of DNA in the presence of 7.5 μl Lipofectamine 3000 (Invitrogen Co) diluited in 250 μl of Optimem (Life Technologies Co). Then washed and incubated 36 h post-transfection.

### Statistics

Unless otherwise specified, all experiments were replicated independently three times and in double or triplicate. Data are given as average ± S.D. Statistical significance was taken for p values <0.05.
